# The effect of *Elymus nutans* sowing density on soil reinforcement and slope stabilization properties of vegetation–concrete structures

**DOI:** 10.1038/s41598-020-77407-1

**Published:** 2020-11-24

**Authors:** Xiangqian Tan, Yongwen Huang, Danwei Xiong, Kun Lv, Fangqing Chen

**Affiliations:** 1 Hubei International Scientific and Technological Center of Ecological Conservation and Management in the Three Gorges Area, China Three Gorges University, Yichang, 443002 Hubei People’s Republic of China; 2 Engineering Research Center of Eco-Environment in the Three Gorges Reservoir Region, Ministry of Education, China Three Gorges University, Yichang, Hubei 443002 People’s Republic of China

**Keywords:** Ecology, Ecology, Environmental sciences, Solid Earth sciences, Engineering

## Abstract

*Elymus nutans* is an herbaceous plant that can be used to restore degraded alpine and subalpine ecosystems. Here, we evaluated how sowing density affects soil reinforcement and slope stabilization properties of vegetation–concrete structures. To investigate the optimal sowing density of *E. nutans* in vegetation–concrete applications for slope protection, six experimental treatments were established with different plant densities: control, I (1100 seeds/m^2^), II (2200 seeds/m^2^), III (3300 seeds/m^2^), IV (4400 seeds/m^2^), and V (5500 seeds/m^2^). Several parameters of plant growth in addition to soil reinforcement and slope stabilization properties were measured in each treatment, as well as the associations among parameters. As density increased, aboveground biomass continually increased, and plant heights, root surface areas, root lengths, and underground biomass all first increased and then decreased. In contrast, tiller numbers and the average root diameter gradually decreased with increasing density. Increased density also resulted in increased maximum water interception levels by aboveground stems and leaves. The maximum water interception by the aboveground stems and leaves was 41.75% greater in the highest density treatment (V) compared to the lowest density treatment (I). However, the enhancement of erosion resistance and soil shear strength first increased and then decreased as density increased, with maximal values observed in the medium-high density treatment (IV). Sowing density was highly correlated with aboveground biomass, plant heights, tiller numbers, and the maximum level of water interception by stems and leaves. Thus, sowing density directly influenced soil reinforcement and slope stabilization properties of aboveground plant components. However, density was not significantly correlated with belowground biomass, root lengths, root surface areas, the enhancement of erosion resistance, and soil shear strengths. Therefore, sowing density indirectly influenced soil reinforcement and slope stabilization of belowground plant components. Following from these results, we suggest that the optimal sowing density of *E. nutans* is approximately 4400 plants/m^2^ in their application within vegetation–concrete structures used for slope protection.

## Introduction

The rapid economic development in southwestern China in recent years has led to the construction of numerous hydropower projects, highways, and railroads that have contributed abundant high and steep anthropogenic slopes in alpine and subalpine regions. The slopes are characterized by severe soil erosion, low stability, and are prone to landslides^1,2^. Further, the slopes lead to various adverse ecological impacts including landscape fragmentation and the loss of species diversity^3^. The active development of protective measures for ecologically restoring slopes hold great promise for slope stabilization, soil and water conservation, and the protection of biodiversity^4,5^. In particular, the use of vegetation in slope protection effectively treats soil erosion and prevents shallow landslides by increasing soil stability on slope surfaces^6,7^. The use of vegetation for soil reinforcement and slope stabilization operates via hydrologic and mechanical effects^8^. The hydrologic effect refers to the reduction of rainfall on slope surfaces through the interception and storage of precipitation by plant stems and leaves that decreases the amount of rainfall on slope soils, and thereby prevents damage to slope stabilization due to excessive precipitation on slopes^9^. The mechanical soil reinforcement effect on slopes by plants refers to the plant root systems that function by increasing soil strength through mechanical properties owing to structural reinforcement by shallow roots and anchorage by deep roots^10,11^.

The Concrete Biotechnical Slope (CBS) is an ecologically based slope protection technology that exhibits good restoration effects. CBS combines the safety protection of side slopes with the vegetation restoration on slope surfaces and have been widely applied for ecological restoration of high and steep slopes in China. The application of CBS not only reduces geological disasters, but also improves the ecological characteristics of the environment^12^. The timely restoration of vegetation coverage is necessary for slope protection projects to fully take advantage of the soil reinforcement and slope stabilization functions of plants^13^. Moreover, planting at an appropriate density will aid in increased soil reinforcement and other slope stabilization properties^14^. However, increased plant densities also increase competition, thereby affecting plant growth. These considerations further impact the properties of soil reinforcement and slope stabilization by vegetation^15–17^. Consequently, evaluating the effects of sowing density on the soil reinforcement and slope stabilization properties in addition to determining optimal sowing densities will provide critical data to inform ecological slope restoration projects.

*Elymus nutans* is a perennial herbaceous plant that belongs to the *Poaceae* family and is mainly distributed in Sichuan, Qinghai, and the Himalayas. The plants are 30–70 cm tall, have erect or geniculate culms, flat leaf blades and pendulous spikes. *E. nutans* sprouts leaves yearly, regrows in April, sets flowers and fruits from July to August, depending on the altitude of its distribution. The whole growth period is about 120 days yearly. Detailed studies of root morphology and mechanisms of cold and drought resistance suggest that the species is an ideal pioneer herbaceous plant for ecological restoration of alpine and subalpine regions of southwestern China^18^. This is especially due to its extensive root system and unique ecophysiological characteristics including resistance to drought, cold, and high salinity^19,20^. However, the properties of *E. nutans* that impact soil reinforcement and slope stabilization have yet to be investigated. Further, the mechanism underlying how planting density in CBS affects vegetation growth and the above characteristics are also unknown. Here, simulated and constructed substrates were used to establish vegetation–concrete structures with different sowing densities of *E. nutans*. Plant growth, soil reinforcement, and slope stabilization parameters, and their associations, were all evaluated among treatments. The specific goals of this study were to (1) investigate aboveground plant growth characteristics including water interception and maximum interception rates among treatments to evaluate the effect of sowing density on critical properties of vegetation–concrete structures for soil reinforcement; (2) investigate belowground plant growth characteristics including erosion resistance and shear resistance among treatments to determine the effects of sowing density on critical properties of vegetation–concrete structures for slope stabilization; (3) determine the associated relationships among sowing density, plant growth characteristics, and properties of soil reinforcement and slope stabilization in order to understand how sowing density influences critical properties of vegetation–concrete structures for soil reinforcement and slope stabilization, and (4) determine the optimal sowing density of *E. nutans* for ecological restoration of slopes in southwestern China.

## Results

Sowing density significantly affected plant growth both aboveground and belowground (*p* < 0.05). Specifically, aboveground biomass continually increased as density increased, while plant heights, root surface areas, root lengths, and belowground biomass all first increased and then subsequently decreased. In contrast, tiller numbers and average root diameters gradually decreased with plant density increases (Table 1). The medium-low density treatment (II) plants exhibited the tallest heights, while the high density treatment (V) plants were the shortest. Plant underground biomass, root surface area, and root length all exhibited maximum values in the medium-high density treatment (IV), with 26.39%, 217.41%, and 135.80% increases, respectively, in those parameters compared to the low density treatment plants. The maximum level of aboveground plant biomass was observed in the high density treatment (IV), while the low density treatments exhibited the lowest level of plant aboveground biomass.

**Table 1:** Physiological characteristics of *E. nutans* under different plant density treatments.

Density treatment	Tillers	Plant heigh (cm)	Aboveground biomass (g/m^2^)	Belowground biomass (g/m^2^)	Root surface area (cm^2^)	Root diameter (mm)	Root length (m)
I	5.13 ± 1.25a	39.38 ± 5.79a	799.18 ± 58.55b	730.83 ± 19.75b	398.95 ± 59.16d	1.16 ± 0.21a	1.62 ± 0.09d
II	5.00 ± 1.31a	40.63 ± 6.41a	847.87 ± 54.73b	806.90 ± 49.45ab	853.73 ± 113.09c	1.08 ± 0.20ab	2.46 ± 0.16c
III	3.93 ± 1.16b	38.42 ± 7.47a	933.52 ± 82.28a	839.40 ± 92.52ab	979.63 ± 212.89bc	0.96 ± 0.14bc	3.34 ± 1.06b
IV	3.20 ± 1.21bc	35.69 ± 6.92ab	961.18 ± 61.81a	923.73 ± 40.89a	1266.31 ± 263.98a	0.93 ± 0.20bc	3.82 ± 0.24a
V	2.87 ± 0.99c	32.84 ± 6.01b	964.83 ± 37.59a	835.90 ± 14.95ab	1083.59 ± 179.49b	0.88 ± 0.15c	3.33 ± 0.23b

**Figure 1:**
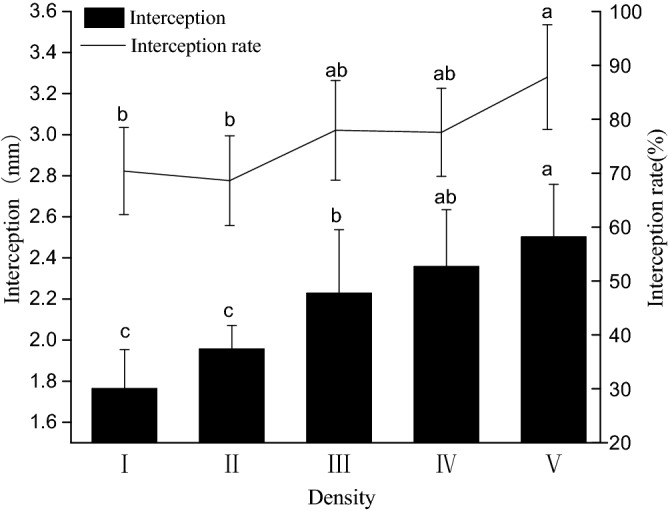
Stem and leaf water interception by *E. nutans* under different plant density treatments. Lowercase letters indicate statistically significant differences (p < 0.05) between different plant density treatments.

The interception of water by *E. nutans* stems and leaves significantly differed with sowing density (maximum interception, F = 5.156, *p* = 0.002; maximum interception rate, F = 6.055, *p* = 0.001) (Fig. 1). The maximum interception and the maximum interception rate both increased with increasing plant density. Specifically, the plants in the high density treatment (V) exhibited the highest maximum interception level and maximum interception rate values of 2.50 ± 0.26 mm and 87.81 ± 9.72%, respectively, which were 42.05% and 24.75% higher than those of plants in the low density treatment.

**Figure 2:**
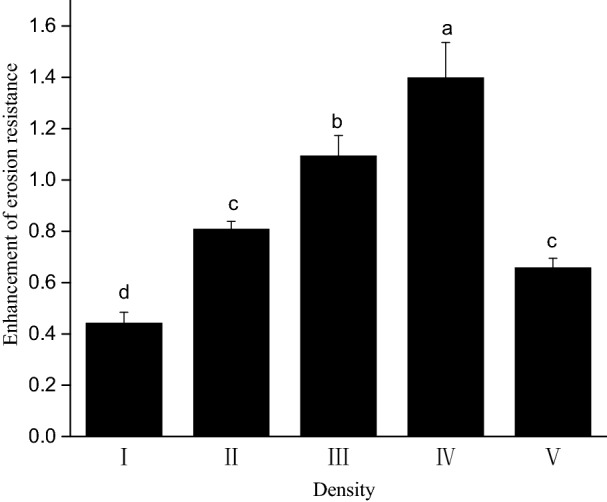
Erosion resistance of *E. nutans* root–soil composites under different plant density treatments. Lowercase letters indicate statistically significant differences (p < 0.05) between different plant density treatments.

The *E. nutans* root system can increase the erosion resistance of vegetation–concrete structures. The average coefficient of enhancement for erosion resistance of the *E. nutans* root–soil system under different sowing densities ranged between 0.31–0.72. Sowing density significantly affected the enhancement of erosion resistance in the root–soil system (F = 192.211, *p* = 0.000). The coefficient of enhancement in erosion resistance first gradually increased and then decreased as sowing density increased (Fig. 2). In particular, the enhancement of erosion resistance in the root–soil system of medium-high density treatment (IV) increased by 136.05% and 65.17% compared to the low density (I) and high density (V) treatments, respectively.

**Figure 3:**
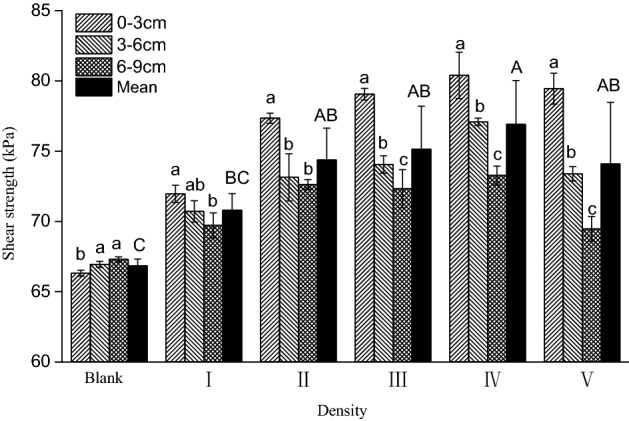
Shear strength of *E. nutans* root–soil composites under different plant density treatments. Lowercase letters indicate statistically significant (p < 0.05) differences between soil layers at different depths within the same plant density treatments. Capital letters indicate statistically significant (p < 0.05) differences between different plant density treatments.

Planting of *E. nutans* can increase the shear resistance of soils. Accordingly, the soil shear strength for all density treatments were significantly higher than those of the control (F = 4.044, *p* = 0.022), with the exception of the low density treatment (I). As sowing density increased, the shear strength of the *E. nutans* root–soil system first increased and then decreased (Fig. 3), with a maximum strength observed in the medium-high density treatment (IV) that was 15.05% greater than that of the control treatment. In addition, the enhancement of shear strength by the *E. nutans* root system differed among soil layers. Specifically, the enhancement effect in the soil surface was significantly higher than that of the middle and bottom soil layers (*p* < 0.05). As sowing density increased, the differences in shear strength among different soil layers also gradually increased.

Sowing density was significantly correlated with plant heights, number of tillers, and aboveground plant biomass (*p* < 0.05) (Table 2). Regarding underground growth characteristics, only the average root diameter was significantly correlated with sowing density. These results indicate that sowing density had a greater effect on aboveground plant growth than belowground plant growth. Nevertheless, aboveground biomass was significantly correlated with root length, root surface area, and root diameter (*p* < 0.05), and the effect of sowing density on aboveground plant growth thus indirectly affects belowground plant growth.

**Table 2:** Correlations among sowing density and physiological indicators of *E. nutans.*

Factors	Density	Plant height	Tillers	Aboveground biomass	Belowground biomass	Root length	Root surface	Root diameter	Water interception	Erosion resistance
Plant height	− 0.911*									
Tillers	− 0.976**	0.931*								
Aboveground biomass	0.949*	− 0.792	− 0.958*							
Belowground biomass	0.550	− 0.353	− 0.618	0.718						
Root length	0.858	− 0.603	− 0.846	0.956*	0.819					
Root surface	0.863	− 0.618	− 0.838	0.928*	0.836	0.984**				
Root diameter	− 0.972**	0.830	0.967**	− 0.994**	− 0.640	0.740	0.879*			
Water interception	0.991**	− 0.873	− 0.978**	0.981**	0.600	0.903*	0.890*	− 0.996**		
Erosion resistance	0.582	− 0.382	− 0.643	0.739	0.999**	0.841	0.860	− 0.646	0.632	
Shear strength	0.649	− 0.338	− 0.636	0.795	0.912*	0.934*	0.943*	− 0.729	0.703	0.924*

*Significantly correlated at the 0.05 level (two-sided); **significantly correlated at the 0.01 level (two-sided).

**Figure 4:**
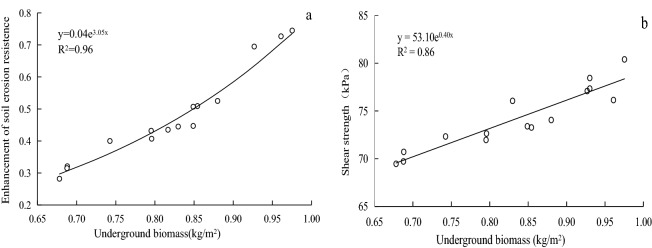
Relationship between slope protection performance and the belowground biomass of *E. nutans.*

In Table 2, a highly significant correlation was observed between sowing density and water interception by stems and leaves (*p* < 0.01). However, sowing density was not significantly correlated to the coefficient of enhancement in erosion resistance or shear strength (*p* > 0.05). These observations indicate that sowing density directly affected the soil reinforcement and slope stabilization properties of aboveground plant components, but not those of the belowground plant components. The coefficient of enhancement in erosion resistance by the root system was highly significantly correlated with belowground biomass (*p* < 0.01), while the shear strength of the root–soil systems was significantly correlated with belowground biomass (*p* < 0.05). In addition, shear strength was significantly correlated with root lengths and root surface areas (*p* < 0.05). Thus, the main factor affecting the coefficient of enhancement in erosion resistance and shear strength was belowground biomass (Fig. 4) including root lengths and root surface areas. Because the correlation between aboveground growth parameters and those of belowground growth were significant, sowing density indirectly influences the coefficient of enhancement in erosion resistance and shear strength.

## Discussion

Aboveground plants intensely compete for resources including light and space as vegetation density increases. Further, plants modify phenotypic characteristics to adapt to environments with heterogeneous density patterns by altering resource distributions among functional traits^21,22^. For example, individual biomass generally decreases as sowing density increases, but the biomass per unit area increases^23^. Some plants also increase their height^24^, but decrease tiller numbers^25^. Here, plant density was significantly and positively correlated with *E. nutans* aboveground biomass. That is, the aboveground biomass per unit area increased as density increased, while individual biomass decreased. However, when density increased beyond the medium density treatment (III), aboveground biomass increased more slowly due to greater competition. Correlation analyses revealed that sowing density was significantly and negatively correlated with plant heights and tiller numbers. Density constraints led to a gradual decrease of tiller numbers to reduce the competition among plants for space, resources, and light. High densities lead to a decreased ability of plants to obtain resources and limits increases in plant heights^26^. Consequently, plant heights first slightly increased in our experiments and then gradually decreased.

Vegetation coverage can protect soil aggregates from destruction by rainfall^27^. In particular, vegetation canopies disperse rainfall into smaller water drops that then fall on sloped surfaces or permeate into soils via stems. Additionally, stems and leaves can absorb and store some precipitation that is later lost to the atmosphere through evaporation and plant transpiration^28^. Previous investigations of vegetation interception of water have mostly focused on forest canopies^29,30^. However, one study by Zhou and David documented that a dense meadow could have an even greater interception effect than forests^31^. Hu et al. (2004) suggested that the interception effect by meadows was mostly determined by aboveground coverage and biomass because the canopy layers are relatively closer to ground surfaces^32^. Generally, higher vegetation coverage and biomass lead to higher water interception. In this study, the maximum water interception by *E. nutans* stems and leaves ranged between 68.61–87.81% among different plant density treatments, which represented significant interception effects by stems and leaves. Pearson correlational analyses further revealed a highly significant association between sowing density and the maximum interception level by stems and leaves. Thus, plant sowing density directly affected soil reinforcement and slope stabilization properties due to aboveground plant characteristics. Further, the maximum interception by *E. nutans* stems and leaves generally increased overall as plant density increased. Lamm and Manges observed differences in interception among different vegetation types, and observed that larger vegetation coverages led to greater overall interception totals. These results are consistent with those presented here^33^.

When plants encounter competition, they usually prioritize the distribution of biomass to organs that are inhibited from receiving adequate resources^34^. Further, when plants are constrained by density, they usually devote more biomass to aboveground growth and reduce biomass input for root growth to avoid shading from neighboring plants and obtain more light^35^. When resources allocated to underground components are limited, root systems undergo “intensive” construction modes, where taproot growth is inhibited and the spatial distribution of roots is decreased to reduce resource input used for root support and transport^36^. In this study, plant density and root diameter were significantly and negatively correlated. As plant density increased, the average root diameter of the root–soil system gradually decreased. Plant density was however not significantly correlated to underground biomass, root surface area, and root length. This result could be explained because these characteristics are not parameters of the root system of individual plants, but rather parameters for the entire root system per unit volume of soil. Consistent with this interpretation, one-way ANOVA analysis indicated that density significantly affected underground biomass, root surface area, and root length. Further, as plant density increased, these parameters first increased then decreased.

The root systems of herbaceous plants are generally densely distributed in the top 30 cm soil layer that is extremely prone to erosion^37,38^. Soil erosion resistance and shear strength are enhanced through the extension, aggregation, and overlap of root systems, and soil stability on slopes is thereby effectively increased^39,40^. The enhancement of root systems on soil erosion resistance and shear strength was related with growth traits and distribution of root systems^41–43^. Because plant density greatly influences belowground plant growth, density also significantly affects belowground plant properties that influence soil reinforcement and slope stabilization^44–46^. Halim and Normaniza showed that high planting densities positively affect the alleviation of slope soil erosion rates^14^. In this study, the *E. nutans* root system in each density treatment greatly increased the erosion resistance and shear strength of vegetation–concrete substrates compared to controls. Nevertheless, the root–soil systems from different density treatments exhibited significant differences in their enhancement of erosion resistance and shear strength. As sowing density increased, the enhancement of erosion resistance and soil shear strength first increased and then decreased, exhibiting maximum values in the medium-high density (IV) treatment. This pattern was also consistent with that of the root system growth parameters. Correlational analysis also indicated that the enhancement of erosion resistance and soil shear strength were significantly associated with belowground biomass. These observations indicate that the enhancement of erosion resistance and soil shear strength were affected by changes in the growth of root systems that were themselves caused by increased plant density. In addition to the above, the *E. nutans* root system exhibited effects on shear strength that differed among soil layers, wherein the shear strength of the top soil layer increased more significantly than those of the middle and bottom soil layers. Consequently, sowing density also induced spatial changes in the shear strength of the root–soil systems. As sowing density increased, the difference in shear strength among different soil layers also gradually increased.

## Methods

*E. nutans* seeds were purchased from a seed supplier with a 1000 grain weight of 3.40 g. Concrete substrates were constructed using the “NB/T 35082-2016 Technical code for eco-restoration of vegetation–concrete on steep slope of hydropower projects” as a reference. The structures were constructed with sandy loam soil, cement, a greening additive, organic matter (dry cow manure), and micro silicon powder mixed at a 100:8:4:7:4 (dry weight) ratio. The micro silicon powder was used to enhance the durability of concrete in an environment with frequent freezing and thawing.

The substrate materials were mixed evenly and then layered in plastic test chambers (34 cm × 26 cm × 12 cm) with drainage holes on the bottom. The layers were then sprayed with water and pressed firmly to achieve a 10 cm thickness. Each test chamber was used as an experimental unit. Referring to the herbaceous sowing density frequently used in the ecological restoration of vegetation concrete^47,48^, six treatments were established with different seed densities: low density (I, 1100 seeds/m^2^), medium-low density (II, 2200 seeds/m^2^), medium density (III, 3300 seeds/m^2^), medium-high density (IV, 4400 seeds/m^2^), high density (V, 5500 seeds/m^2^), and a control plot. Six replicates were used for each treatment comprising a total of 36 experimental units. Seeds were weighed, sufficiently mixed with substrate materials, and evenly dispersed into their respective test chambers. The test chambers were then slightly shaken and pressed firmly. The chambers were placed at a 45° angle to mimic the growth environment of slopes. Water addition was conducted daily to ensure the soil was moist.

Six months after sowing, the soils in each test chamber were saturated with water and randomly sampled from the soil substrate in each unit using a cutting ring (8 cm diameter, 10 cm depth). Each treatment consisted of six samples, with total collection of 36 samples.

The aboveground components of plants in each treatment were removed along soil surfaces. Plant heights, tiller numbers, and the fresh weight of each aboveground sample were then measured. Water interception by stems and leaves was measured using the simplified water absorption method^32^. Briefly, stem and leaf samples were completely submerged in water for 5 min, gently removed, and water was allowed to drip completely off by gravity. The weights were then measured again. The maximum water interception level and maximum interception rate were calculated to represent the overall water interception by stems and leaves. The maximum interception rate was determined as the percentage of water absorbed by plant stems and leaves within the fresh weight of stems and leaves (Eq. 1). The maximum interception was determined as the thickness of the water layer and was calculated using the amount of water absorbed by stems and leaves per unit area (Eq. 2).

1 $${\text{R}}_{{{\text{max}}}} = ({\text{W}}_{2} - {\text{W}}_{1} )/{\text{W}}_{1} \times 100\%$$

2 $${\text{W}}_{{{\text{max}}}} = {\text{R}}_{{{\text{max}}}} \times {\text{M}}_{1} /10$$

Here, R_max_ is the maximum interception rate (%), W_1_ is the weight of stem and leaf samples before water absorption (g), W_2_ is the weight of stem and leaf samples after water absorption, W_max_ is the maximum interception (mm), and M_1_ is the fresh weight of stems and leaves per unit area (t/hm^2^).

To measure aboveground biomass, the aboveground components of each sample were placed in a baking oven at 105 °C for 20 min to deactivate the enzymes. The samples were then dried at 80 °C until a constant weight was achieved, followed by measurement of biomass.

Erosion resistance and shear resistance were measured using root–soil system samples from each treatment. The erosion resistance of the underground systems was measured using the method of collapse resistance in static water^37,38^. Erosion resistance was calculated using the equation below:

3 $${\text{Ce }} = {\text{V}}_{0} /{\text{V}}_{2} = ({\text{V}}_{1} - {\text{V}}_{2} )/{\text{V}}_{1}$$

where Ce is a coefficient corresponding to the reduction of the soil disintegration rate due the root system and thus reflects enhancement of erosion resistance in the root system; V_1_ is the soil disintegration rate of the control soil; V_2_ is the soil disintegration rate of soils with roots; and V_0_ is the reduction of the soil disintegration rate due to the presence of the root system. The soil disintegration rate was expressed as the weight of disintegrated soil saturated with water per unit time: V = M/T = (M_1_ − M_2_)/t (g/min). Here, M_1_ is the initial soil weight, M_2_ is the soil weight after 30 min, and t is time. The detachment rate of samples was tested using the method described by Burylo et al.^38^. Each sampled soil column was weighed, and then placed vertically on a steel wire mesh and completely immersed in static water to disintegrate for 30 min. The residual soil column was taken out and weighted. The specific disintegration time were recorded and used to calculate the soil disintegration rate when a whole soil column detached within 30 min. Three replicate measurements were taken for each treatment.

The shear resistance properties of the underground systems were measured using a ZJ Strain Controlled Direct Shear Test Apparatus^49,50^. The root–soil system samples were vertically separated into three layers: 0–3 cm, 3–6 cm, 6–9 cm, to determine shear resistance. Test samples were trimmed and placed in a shear box (6 cm diameter, 2 cm depth), and a horizontal force was applied under 100 kPa vertical pressure. The shear strength was then measured when the soil sample failed, as calculated with the following equation:

4 $$\tau = {\text{KR}}$$

where τ is the shear strength (kPa); K is the quantum ring rate coefficient of determination (2.50 kPa/0.01 mm); and R is the unit of the dial test indicator readings (0.01 mm). Three replicate measurements were taken for each layer. The shear strengths of the three soil layers in each treatment were then averaged to represent the shear strength of the root–soil system. A total of nine samples were measured for each treatment.

After measurement of erosion resistance and shear strength, the entire root system from every root–soil sample was carefully cleaned and separated. A root system imaging system (WinRHIZO root analysis system, Regent Instruments Inc.) was then used to measure the average root diameter, total root length, and root surface area within each root–soil system. Finally, the root systems from every sample were dried at 80 °C until achieving a constant weight in order to measure underground biomass.

Sowing density of *E. nutans* was used as the independent variable, while plant growth parameters and soil reinforcement and slope stabilization parameters were dependent variables in one-way ANOVA analyses. Duncan's multiple range test was used to analyze the significance of differences among each treatment and evaluate the effect of sowing density on plant growth, soil reinforcement properties, and slope stabilization properties. Further, Pearson correlation coefficient analyses were conducted with sowing densities of *E. nutans*, plant growth characteristics, soil reinforcement, and slope stabilization to evaluate their associations. All statistical analyses were conducted in the SPSS 19.0 software program.

## Data Availability

Raw and processed data for individual cruises, along with details of the processing, can also be obtained upon reasonable request to corresponding author (fqchen@ctgu.edu.cn).
